# Cultural Contexts of Ebola in Northern Uganda

**DOI:** 10.3201/eid0910.020493

**Published:** 2003-10

**Authors:** Barry S. Hewlett, Richard P. Amola

**Affiliations:** * Washington State University, Vancouver, Washington, USA; † Ministry of Health, Adjumani, Uganda

## Abstract

Technical guidelines for the control of Ebola hemorrhagic fever (EHF) indicate that understanding local views and responses to an outbreak is essential. However, few studies with such information exist. Thus, we used qualitative and quantitative methods to determine how local residents of Gulu, Uganda, viewed and responded to the 2000–2001 outbreak of EHF. Results indicated that Acholi people used at least three explanatory models to explain and respond to the outbreak; indigenous epidemic control measures were often implemented and consistent with those being promoted by healthcare workers; and some cultural practices amplified the outbreak (e.g., burial practices). However, most persons were willing to modify and work with national and international healthcare workers.

Many emerging disease specialists are sensitive to and acknowledge the potential importance of social science in disease control, but seldom is this perspective considered when organizing response efforts. In part, this situation exists because so little research in this area has been conducted. The special issue on Ebola in The Journal of Infectious Diseases (*1*) does not include any articles on the behavioral aspects of the disease. However, World Health Organization (WHO) technical guidelines for responding to Ebola hemorrhagic fever (EHF) state that, in conducting epidemiologic surveillance, “Special attention must be given to the actual perception of the outbreak by the community. In particular, specific cultural elements and local beliefs must be taken into account to ensure proper messages, confidence, and close cooperation of the community” (*2*).

We describe the first systematic sociocultural study of an outbreak of EHF. The outbreak occurred in several locations in northern Uganda in 2000 to 2001. We conducted this research in villages and neighborhoods in and around Gulu during the last month of the outbreak. The field study aimed to: 1) describe local explanatory models of EHF; 2) provide understanding of topics of concern to WHO (i.e., burial practices, patients’ fear of going to the hospital, the role of traditional healers in disease transmission); and 3) identify local and international beliefs and practices that enhanced or were detrimental to the control of EHF. An “explanatory model” refers to a person’s or culture’s explanations and predictions regarding a particular illness. Some of the questions asked when trying to understand an explanatory model include: How do persons refer to the illness? How do they explain it (i.e., cause)? What do they see as appropriate treatments? What do they do to prevent the illness? Patients, physicians, healthcare workers, and local residents in different parts of the world each have explanatory models for different illnesses. Providing care and treatment for a particular disease is often based on negotiating these different models.

## Background

The 2000–2001 Uganda outbreak was one of the largest EHF outbreaks to date, with 425 presumptive cases and 224 deaths (case-fatality rate 53%). Most patients were women (269 or 63%). The earliest reported presumptive case had disease onset on August 30, 2000, and the last case began on January 9, 2001 (*3*).

The Gulu EHF outbreak was relatively unusual in comparison to other recent EHF outbreaks (e.g., Democratic Republic of Congo [DRC], Gabon) in that the disease affected primarily one ethnic group, the Acholi, and most of the district medical staff and decision makers (e.g., district medical officer, director of health education) were also from this ethnic group. Also, most (60%) EHF cases occurred in the urban area of Gulu town. Gulu District has approximately 470,000 people, primarily Acholi, and 60% of the population live in protected villages because of rebel activity.

Most Acholi are agro-pastoralists and have a social organization strongly influenced by patrilineal descent and patrilocal postmarital residence (*4*). Other researchers have written about Acholi and other Nilotic peoples’ health beliefs (*5*–*7*), but none has described cultural responses to epidemic diseases.

## Methods

Qualitative and quantitative methods were used. The first 2 weeks of the research emphasized open-ended and focus group interviews as well as document review (e.g., health education materials, reports). The last few days emphasized the development of systematic questionnaires. Open-ended interviews were conducted with the following: 1) 10 persons and 4 focus groups in villages or neighborhoods with large numbers of early cases of EHF; 2) 8 persons and one focus group with survivors of EHF (both healthcare workers and community members); 3) four focus groups with male and female elders (two meetings with each gender); 4) 3 persons and two focus groups of children; 5) 4 persons and two focus groups with healthcare workers responsible for the isolation unit and counseling survivors; and 6) 4 persons and one focus group with traditional healers. Focus group meetings usually had 5–8 participants, with the exception of the survivor focus group meeting, which had 35 participants.

Questionnaires were administered to 85 Gulu High School students 15–21 years of age (all members of three senior classes; 33 men, 52 women). Precoded questionnaires were administered to 49 adults in Gulu (25 women, 24 men; an adult from every third house from two randomly selected Gulu neighborhoods) and 60 EHF survivors (22 men, 38 women; all survivors were located through a survivors’ organization). We also examined existing documents, such as field reports and health education materials used in the outbreak (e.g., posters, brochures, music cassettes, videos).

## Results

### Explanatory Models

Table 1 summarizes three primary explanatory models identified by Acholi. The third model is biomedical. Biomedical models have existed in the area for >100 years, and all Acholi know these models well and use them often. In the early phases of the outbreak, many families, thinking the disease was a bacterial infection or malaria, turned to tetracycline or chloroquine. Most early case-patients went to the hospital seeking biomedical treatment. The biomedical model for EHF was introduced in late October by the Ugandan Ministry of Health. The health education program was multidimensional (e.g., posters, radio shows, videos, brochures) and transmitted this model effectively. However, by the time the EHF biomedical model was introduced, local people had already used two other indigenous explanatory models.

**Table 1:** Explanatory models for Ebola hemorrhagic fever (EHF) among the Acholi^a^

Terms	*Yat*	*Gemo*	Disease of contact; Ebola
Description	“Medicine” or substance that enters the body and causes illness	Bad spirit that comes suddenly and rapidly and effects many people	EHF, biomedical description
Signs and symptoms	Starts with pain inflammation but can have many other signs in later stages	Mental confusion, rapid death, high fever	High fever, vomiting, headache
Causes	Bad “medicine” (poison) goes into body	Lack of respect for *jok*, sometimes no reason	Filovirus, but host reservoir unknown
Transmission	Step on it, eat it, catching it, somebody sends, just looking at a person	Physical proximity, easy for *gemo* to catch you	Physical contact with bodily fluids of patients
Pathophysiology	Inflammation and pain in area touched by or location of *yat*	Attacks all of body	Damage to major organs
Treatment	*Tak—*techniques of healers who use their *jok* to identify and remove *yat* from body or environment	Talk to *jok* via traditional healer, give whatever wants, gifts of food to *jok*	None, hydrate (ORS), control vomiting
Prevention and control	Protective bracelets	See protocol in text, *chani labolo, ryemo gemo*	Do not touch patients, barrier nursing
Prognosis	Good if removed from body; otherwise death	Not good, no cure	Not good, no cure
Risk groups	Very smart, successful, salaried people; anybody	Caregivers close to patients (women), families that do not respect *jok*, families that do not follow protocol	Unprotected healthcare workers, caregivers of patients, people that wash or touch dead victims
Political	Infected troops returning from DRC sent to Gulu	Infected troops returning from DRC sent to Gulu	Infected troops returning from DRC sent to Gulu

^a^ORS, oral rehydration salts; DRC, Democratic Republic of Congo.

Both of these models require an understanding of the concept of *jok*, which is common to many Nilotic-speaking peoples, including the Acholi. *Jok* are spirits or gods (*8*). Many different types of *jok* exist; they have names and reportedly are often found near bodies of water, mountains, and natural salt licks for cattle. *Jok* are generally benevolent, as they provide and control resources, but they can also cause harm if they are not respected. Deference to and respect for others are central values in Acholi life, and spiritual life reflects and reinforces these values. These spirits are like elders in the community; the Acholi listen to what they have to say, do what they say without question, and give them gifts to show respect.

Traditional healers (*ajwaka*), who are primarily women, obtain their powers to heal from specific *jok* that they have acquired through time. Most healers acquire as many as 10 such spirits during their lifetime. Each spirit has a name and a specific kind of knowledge (e.g., treatments for mental confusion or infertility).

At first, many persons treated the symptoms of EHF as a regular illness and sought a variety of both biomedical (i.e., malarial drugs or antibiotics) and indigenous cures (i.e., herbs, traditional healers). In late September 2000, the heads of families in neighborhoods with many deaths asked a traditional healer to locate poisons (*yat*) in and around the lineage household that might be causing the illness and death (Table 1, first explanatory model). The healers used their *jok* and special spears to locate poisoned objects (e.g., roots, bones) in the neighborhood. The healer’s *jok* was also called on to communicate with the spirit associated with a particular poison and to determine if burning the object and sacrificing goats and sheep was necessary to demonstrate respect. Once the poison was removed and respect demonstrated, the healers said the deaths would stop. But the deaths continued. *Yat* removal is not cheap; each EHF-affected family paid 150,000 Ugandan shillings (U.S.$88), four to five goats or sheep, and one chicken (about half the annual income for a rural Acholi family). The families pointed out that in addition to the enormous loss of loved ones, an incredible amount of family assets was lost in trying to treat the ill.

In early October, residents began to realize that this outbreak was more than a regular kind of illness and began to classify it as *two gemo* (two [illness] gemo [epidemic]), the second explanatory model in Table 1. *Gemo* is a bad spirit (type of *jok* that comes suddenly and causes a mysterious illness and death in many people within a very short period of time). *Gemo* reportedly comes like the wind in that it comes rapidly from a particular direction and affects many people, but the wind itself does not necessarily bring it. Acholi have experienced other types of *gemo* (e.g., measles and smallpox ). Forty-nine of 50 adults interviewed indicated a belief that Ebola was a type of *gemo*. The term *two gemo* was also used in health education posters and music.

*Gemo* is said to be mysterious in that is just comes on its own, but several people indicated that it comes because of lack of respect and honor for the gods. Elders indicated that in the past, lack of respect for *jok* of *tura* (hills, mountains, bodies of water) was the major cause of *gemo*. People talk about *gemo* catching you, so if someone is close to a person with *gemo* it is easier for *gemo* to catch you. Once an illness is identified as *gemo*, a protocol for its prevention and control is implemented that is quite different from the treatment and control of other illnesses.

When an illness has been identified and categorized as a killer epidemic (*gemo*), the family is advised to do the following: 1) Quarantine or isolate the patient in a house at least 100 m from all other houses, with no visitors allowed. 2) A survivor of the epidemic should feed and care for the patient. If no survivors are available, an elderly woman or man should be the caregiver. 3) Houses with ill patients should be identified with two long poles of elephant grass, one on each side of the door. 4) Villages and households with ill patients should place two long poles with a pole across them to notify those approaching. 5) Everyone should limit their movements, that is, stay within their household and not move between villages. 6) No food from outsiders should be eaten. 7) Pregnant women and children should be especially careful to avoid patients. 8) Harmony should be increased within the household, that is, there should be no harsh words or conflicts within the family. 9) Sexual relations are to be avoided. 10) Dancing is not allowed. 11) Rotten or smoked meat may not be eaten, only eat fresh cattle meat. 12) Once the patient no longer has symptoms, he or she should remain in isolation for one full lunar cycle before moving freely in the village. 13) If the person dies, a person who has survived *gemo* or has taken care of several sick persons and not become ill, should bury the persons; the burial should take place at the edge of the village.

From a biomedical perspective, this protocol constitutes a broad-spectrum approach to epidemic control. Isolation and identification of the patient’s home and village were emphasized by all groups interviewed, but sexually transmitted and foodborne transmissions were also frequently listed. Elders were adamant that this protocol existed before the arrival of Europeans in the late 1800s. Although historic research is needed to verify this claim, the facts that an indigenous term (*gemo*) is associated with the behaviors, the belief is integrated into the religious system (*jok*), and the protocol is common knowledge to children who do not learn it in school suggest that many rules existed in pre-Colonial times.

Several other ways exist to try to control *gemo*, including driving it away to the Nile by noisemaking (*Ryemo gemo*). This procedure was conducted several times during the outbreak and is conducted every December 31 to chase away any potential *gemo* before the New Year begins. Another local custom is *Chani labolo,* which consists of wearing a dried banana leaf bracelet for 3 days (for men) or 4 days (for women) to protect and chase away *gemo*. Some healers have *jok* that is supposedly specific for *gemo*, and three of the four traditional healers interviewed indicated their *jok* told them about the impending *gemo* before it arrived (i.e., back in August 2000).

Most local residents saw a political dimension to the explanatory models of EHF (Table 1). Many felt that EHF came from infected Ugandan soldiers returning from DRC. Residents felt that the current government has little interest in the North so when Ugandan soldiers became infected in the DRC, the decision was made to send them to military bases in Gulu. Although some of the first female victims of EHF had relationships with men in the military, existing epidemiologic evidence does not support the DRC origin hypothesis. The origin of this outbreak is not known. Political dimensions to disease, killer epidemics, in particular, are common. The first author’s visit to Gabon during the 1996 EHF outbreak indicated that local people believed that the French military, which had military exercises in the area just before the outbreak, were partially responsible for introducing the disease. In addition, the 2003 Congo outbreak was linked, in part, to activities of Euro-Americans conducting research in a national park there.

Many informants and healthcare workers indicated that fleeing the village or neighborhood was common, in particular in locations with the highest number of infected persons. Fleeing is not an explicit part of the explanatory models but makes some sense because the *gemo* or biomedical models indicate the illness is a rapid killer transmitted by close contact with infected persons.

Most persons involved in this outbreak were familiar with all three explanatory models and did not necessarily see them as contradictory. Healthcare workers emphasized the biomedical model, but many Acholi healthcare workers participated in *ryemo gemo* when it passed through the community. Some persons and villages turned to the *yat* and *gemo* explanatory models but did not hesitate to purchase tetracycline and other medicines to treat cases of EHF. The first two explanatory models may seem strange to international health workers, but they reflect a holistic and social view of illness common to many people in the world. Acholi are aware of the biomedical model but view illness as having social, spiritual, and biological dimensions. The epidemic control protocol is a good example. Family members refrain from sex and quarreling to show respect to *jok* (spiritual) and increase family harmony and peace (social).

### Issues of Concern

#### Funerals and Burials

National and international healthcare workers were concerned that burial practices contributed to the amplification of EHF. A brief study indicated that once a person died, his or her paternal aunt (father’s sister) was called to wash and prepare the body for burial. If the father did not have a sister, an older woman in the victim’s patriline was asked to prepare the body. Generally, the woman removed the clothes from the body, washed the body, and dressed the deceased in a favorite outfit. At the funeral, all family members ritually washed their hands in a common bowl, and during open casket all were welcome to come up to deceased person and give a final touch on the face or elsewhere (called a love touch). The body was then wrapped in a white cloth or sheet and buried. The person was buried next to or near their household. This practice is the normal system of burial.

However, when disease is classified as *gemo,* burial practices change*.* The body is not touched and is buried outside or at the edge of the village. The designated caregiver, someone who has survived the outbreak or an older woman, is responsible for washing and preparing the body for burial.

Various activities associated with burial practices contributed to transmission of EHF (Table 2). Washing the body was a possible means of infection for women only, while a touch was a more common means of infection among men. The fact that 63% of the survivors in this study had their first symptoms in October implies that they probably became infected before laboratory tests confirmed EHF and before the disease was designated as a type of *gemo* in many communities. Caregiving, especially by women, contributed substantially to many cases, which explains, in part, why 67% of all presumptive EHF cases in Uganda were in women.

**Table 2:** How survivors thought they contracted Ebola hemorrhagic fever (EHF)^a^

How survivors felt they acquired Ebola	%	
	Men (n=22)	Women (n=38)
Washing body of EHF victim	0	21
Love touch	32	11
Transporting EHF patient to hospital	5	16
Caregiving of EHF patient	27	53

^a^More than one response possible per informant.

WHO was also concerned that local persons were not coming to the hospital when symptoms first emerged. Healthcare workers theorized that patients were afraid of being buried at the airfield if they died. Persons were running and hiding when the ambulance arrived to take them to hospital. Later interviews indicated, however, that the airfield burial was not the problem. As described in the protocol, once an illness is identified as a killer epidemic, burial at the edge of the village is expected. Rather, sources indicated, many persons ran from the ambulance and did not seek treatment quickly because they feared they would never see their family once they were admitted to the hospital. This fear is common in many parts of central Africa but was especially pronounced in Gulu hospitals because bodies were placed in body bags and taken to the airfield to be buried without relatives being notified. Relatives were not always around at the time of death, and healthcare workers were required to dispose of the body as quickly as possible. The anger and bad feelings about not being informed were directed toward healthcare workers in the isolation unit. This fear could have been averted by allowing family members to see the body in the bag and allowing family members to escort the body to the burial ground.

### Traditional Healers

The term traditional healers is used here because it is commonly used by WHO and other international agencies. In the Gulu area, however, such healers are often referred to as witchdoctors. Both terms misrepresent the nature of what they do. The term traditional gives the impression that their practices have not changed since time immemorial, when, in fact, such healers are always changing their practices. For instance, as mentioned, they no longer suck out *yat* with their mouths because some healers who did so contracted and died from HIV/AIDS. Today such healers use a local sponge or type of grass to extract *yat*. The term witchdoctor is even more misleading because witches (called night dancers or *lajok*) are relatively uncommon in this area by comparison to the Bantu-speaking areas to the south, and few healers know how to treat witchcraft. Indigenous healer may be a more appropriate term.

Before this study was conducted, WHO and other international and national health workers felt that traditional healing practices of some healers led to the amplification of the outbreak. A female healer and some of the earliest EHF patients were often mentioned as examples. In September, a healer traveled from Gulu town to her rural village a few days after treating a known EHF patient. The healer became ill and reportedly treated patients by cutting and sucking poisons, such as *yat,* from ill patients, and thus infecting her patients with her bodily fluids. The healer died, and >10 deaths were subsequently associated with her healing. But village sources indicated that she did not treat people in the rural village and she did not have any of her healing tools (e.g., spear and rattle) because rebels in the rural areas kill healers caught with these implements (rebels view the work of healers as contrary to the ways of God). Rather, the healer infected many people because she was a prominent and powerful healer. Consequently, when she became ill in the rural area, many people assisted in her care, several different persons slept with her during the night to watch after her, and once she died, several persons assisted in the traditional washing of her body. This case occurred early in the outbreak, and misunderstanding led health authorities to ban all traditional healing. Traditional healers were stigmatized, which may have been unfortunate as all those we interviewed wanted to help in control efforts. As mentioned above, healers rarely cut the skin to remove *yat* or to insert medicines or herbs because of HIV/AIDS health education programs and the loss of several healers to that infection.

### Stigmatization

Independent research on stigmatization was conducted by Kabananukye (*9*) so only a limited number of results from our study will be described. Adults were asked when they would feel comfortable touching a person who survived Ebola: on the day of hospital release, after 2 weeks, after 1 month, or after >1 month. The most common response (49%) was 1 month after hospital release. This response is consistent with the epidemic control protocol described previously, but many survivors experienced stigmatization long after this 1-month period, in part, because they continued to experience other illnesses (e.g., vision problems, fatigue, leg pains).

Many survivors experienced intense stigmatization. Some were not allowed to return home, many had all their good clothes burned, and some were abandoned by their spouses. Their children were told not to touch them, and wives were told to go back to their home villages. The discrimination also extended to family and village members. For instance, community members from one of the first rural villages affected were regularly turned away at the marketplace and watering hole. One man eventually committed suicide, in part, because he had lost his wife to EHF but also reportedly because of the stress of rejection, harassment, and discrimination in public because of his association with EHF. The survivors’ questionnaires suggested that women experienced somewhat greater stigmatization than men. Table 3 summarizes some of these findings.

**Table 3:** Ways and locations in which Ebola survivors felt stigmatized

Locations in which survivors felt stigmatized	% of yes responses	
	Men (n=22)	Women (n=38)
Feared by others when you returned to the community	55	82
Rejected at market or store	36	58
Rejected at well or borehole	32	58
Rejected when walking through neighborhood	55	76

## Discussion

Several limitations apply to our study: 1) it was conducted within a relatively short period of time (16 days); 2) researchers were not allowed to live in a village as participant observers because of political insecurity, and 3) the study was conducted at the end of the outbreak. Given these limitations, the study nevertheless provided useful data for control efforts.

Fred Dunn (*10*), a physician and anthropologist, developed a simple framework for integrating anthropologic work into disease control efforts. The framework is useful because it emphasizes identifying both health-enhancing and health-lowering beliefs and practices of both the local, national, and international communities. Many sociocultural studies tend to focus only on how local beliefs and practices amplify the disease (e.g., how traditional burial practices contribute to disease transmission); little attention is given to how local peoples’ beliefs and practices might contribute to control efforts. Many models also do not examine the beliefs and practices of the biomedical community. The data from this limited study are placed in Dunn’s framework in Tables 4 and 5. These data indicate that local, national, and international actions contributed to the control of this outbreak. All of the health-lowering activities in the community were targeted for change by health educators. Most of the health-lowering activities of the national and international teams were recognized shortly after they occurred. Many beliefs and practices are neutral in that they do not help or hinder transmission of EHF. For instance, chasing away *gemo* by using *ryemo gemo* or *chani labolo* did not clearly help or hinder disease transmission.

**Table 4:** Community beliefs and practices that enhanced and lowered health of some persons during Gulu Ebola hemorrhagic fever outbreak

Health enhancing	Health lowering
Indigenous protocol for epidemics (see text)	Some aspects of burial and funeral practices: washing of body, dressing the body, love touches, and ritual washing of hands in common bowl of water
Elders sought to help organize the community	Transporting sick or dead by bike, cart, or other means
	Some aspects of traditional healing practices, such as cutting of body to insert medicines

**Table 5:** Beliefs and practices of the national and international healthcare professionals that enhanced and lowered health of some persons during Gulu Ebola hemorrhagic fever (EHF) outbreak

Health-enhancing beliefs and practices	Health-lowering beliefs and practices
Most national government health workers and decision makers spoke local language and had an understanding of local cultures	Unintended consequences of WHO^a^ health education video: burning of houses of survivors
Establishment of isolation unit and use of barrier nursing	Taking bodies to burial ground before family members could verify the death. This practice led to sick persons hiding from family and health workers; family members being afraid to take sick persons to hospital; persons running away from the ambulance; and stories of Europeans selling body parts
Providing gloves and bleach to local communities	Omitting traditional healers from control efforts; they were ready and willing as a group to help mobilize the community
Medical care of Ebola victims including rehydration, control of vomiting, other drugs/medications	Early stages only: 1) nurses and healthcare nurses lacked training about barrier nursing, protective gear, and education about the transmission and nature of the disease; 2) lack of transport for sick patients; 3) international health workers not familiar with naming, kinship system, household organization of local communities
Multidimensional health education	Taking blood samples for research only or blood taken without reporting results back to persons or communities’ increased distrust of healthcare workers
Suspension of the following activities: handshaking upon greeting, cutting by traditional healers, schools, discos, public funerals, traditional beer drinking	International team members conducting EHF studies for research only. This diverted time and energy from control efforts
Diagnostic laboratories for Ebola	
Ambulances to transport patients to hospital to isolate	
Reallocation of tasks of health workers to focus on EHF	
Use of mobile teams to follow all contacts and provide health education, support for survivors and impacted families	

^a^WHO, World Health Organization; EHF, Ebola hemorrhagic fever.

Most national and international physicians, nurses, and healthcare workers are supportive of sociocultural studies, but most do not have the time, especially in outbreak situations, or tool kits to conduct the kinds of studies that might be useful. In the short term, social scientists can contribute to: 1) epidemiologic studies (how to identify persons, personal naming systems, kinship terms, clan names); 2) doctor-patient relations (international healthcare workers understanding of local explanatory models); 3) control efforts (cultural practices and beliefs that may be amplifying outbreak, identifying and mobilizing existing cultural institutions); and 4) health education (which cultural practices and beliefs to build upon, where to focus change).

Many national and international healthcare workers tend to view cultural practices and beliefs as something to overcome, and certain cultural burial practices (washing the body and love touches) did initially amplify EHF in Uganda. However, once people realized that EHF killed rapidly and classified it as *gemo*, a different set of cultural practices and beliefs were implemented. One reason the health education program worked so well was that it was in many ways consistent with indigenous epidemic control measures (isolation, suspension of greetings, dances, public funerals). Even the burying of victims at the airfield, while a bit dramatic for some, was consistent with burying *gemo* victims outside or at the edge of the village

Sensitivity to cultural factors associated with the control of chronic infectious and parasitic disease has increased in the past 20 years, but little attention has been given to cultural factors associated with emerging infectious diseases, especially diseases such as EHF that cause rapid death. The urgent context of these outbreaks often leads to the neglect of local people’s feelings and knowledge. The general impression is that, without Western intervention, the epidemic would kill hundreds and spread to all parts of the world; local practices and beliefs are perceived only as amplifying the outbreaks. Our study was the first systematic sociocultural study of EHF. It showed that some cultural practices did indeed amplify the outbreak. However, an important finding was that local people have beliefs and practices in place that can be useful to control rapid epidemics, such as EHF, with high fatalities. Because local people have lived with high mortality rates and serious epidemics for some time, their knowledge may be useful to national and international teams in their efforts to control emerging diseases.
